# Antioxidant and Anti-Inflammatory Activities of Hydrolysates and Peptide Fractions Obtained by Enzymatic Hydrolysis of Selected Heat-Treated Edible Insects

**DOI:** 10.3390/nu9090970

**Published:** 2017-09-02

**Authors:** Ewelina Zielińska, Barbara Baraniak, Monika Karaś

**Affiliations:** Departament of Biochemistry and Food Chemistry, University of Life Sciences in Lublin, Skromna Str. 8, 20-704 Lublin, Poland; barbara.baraniak@up.lublin.pl (B.B.); monika.karas@up.lublin.pl (M.K.)

**Keywords:** antioxidant activity, anti-inflammatory activity, digestion, edible insects, entomophagy, peptide fractions

## Abstract

This study investigated the effect of heat treatment of edible insects on antioxidant and anti-inflammatory activities of peptides obtained by in vitro gastrointestinal digestion and absorption process thereof. The antioxidant potential of edible insect hydrolysates was determined as free radical-scavenging activity, ion chelating activity, and reducing power, whereas the anti-inflammatory activity was expressed as lipoxygenase and cyclooxygenase-2 inhibitory activity. The highest antiradical activity against DPPH^•^ (2,2-diphenyl-1-picrylhydrazyl radical) was noted for a peptide fraction from baked cricket *Gryllodes sigillatus* hydrolysate (IC_50_ value 10.9 µg/mL) and that against ABTS^•+^ (2,2′-azino-bis(3-ethylbenzothiazoline-6-sulfonic acid) radical) was the highest for raw mealworm *Tenebrio molitor* hydrolysate (inhibitory concentration (IC_50_ value) 5.3 µg/mL). The peptides obtained from boiled locust *Schistocerca gregaria* hydrolysate showed the highest Fe^2+^ chelation ability (IC_50_ value 2.57 µg/mL); furthermore, the highest reducing power was observed for raw *G. sigillatus* hydrolysate (0.771). The peptide fraction from a protein preparation from the locust *S. gregaria* exhibited the most significant lipoxygenase and cyclooxygenase-2 inhibitory activity (IC_50_ value 3.13 µg/mL and 5.05 µg/mL, respectively).

## 1. Introduction

Entomophagy is one of the most popular trends of human nutrition in Europe. Insects are consumed as a whole and they are a potential ingredient of food preparations providing nutrients, such as protein, fatty acids, or microelements [1]. Foods based on insects are mostly processed, but some people do also eat raw, whole insects. Traditionally, insects are often consumed quickly after harvesting. Traditional methods of preservation include sun-drying and heating. Nowadays, the most popular processing methods are drying, pulverizing or grinding, and heating (e.g., baking, boiling, and canning) or preserving by freeze-drying or vacuum packing. Insects can also be processed into pastes and powders. In most Western countries, however, people view entomophagy with disgust and associate eating insects with primitive behaviour, but insect proteins, fats, and chitin can be isolated before use in food products as well. This method may contribute to the consumption of insects because their appearance is not recognizable anymore [2,3,4]. Nevertheless, the interest for insects as food in the Western world continues to increase. With time, in some European countries, insects were given the position of sophisticated gourmet dishes, offered in restaurants, presenting them as much more attractive [5].

Consumption of insects may also enrich the human diet in bioactive components, for example biologically-active peptides [6]. Bioactive peptides released during gastrointestinal digestion, or by previous in vitro protein hydrolysis, are small compounds with beneficial biological activity, for example antihypertensive, antioxidant, anti-inflammatory, or hypocholesterolemic activity [7,8,9,10]. Bioactive peptides have been studied mainly in milk and derived products (cheese, yogurt), and plant proteins, such as soy, rice, bean, and chickpea [7,11,12,13].

Free radicals are constantly generated from normal metabolic processes in the body, but oxidative stress occurs as a result of imbalance between the generation of reactive oxygen species (ROS) and the ability of cells to neutralise them. Oxidative stress is associated with the development of so-called “civilization diseases”, such as cancer, stroke, myocardial infarction, or inflammation, as well as the degenerative process associated with aging, including Parkinson’s and Alzheimer’s diseases [14,15]. Consumption of foods rich in antioxidants plays an essential role in the prevention of these diseases. Several studies have shown that antioxidant and anti-inflammatory peptides have protective effects against ROS and may contribute to a significant reduction of the level of oxidative stress [6,11,12,16]. Inflammation is a complex biological process, occurring through a variety of mechanisms and leading to changes in local blood flow and the release of several molecular mediators. The dual 5-LOX/COX inhibitors induce an enhanced anti-inflammatory effect and act by blocking the formation of both prostaglandins and leucotrienes without affecting lipoxin formation. In addition, such combined inhibition prevents damage to the gastrointestinal mucosa [17]. However, the inhibition of COX-2/5-LOX has been reported to promote the other pathway of arachidonic acid metabolism as a compensatory mechanism. Considering the similarities of COX-2 and 5-LOX, the dual inhibition thereof to obtain more efficient and safer agents for human cancer treatment is promising, albeit the exact mechanism has not been fully illustrated [18].

Given the wide range of edible insect species, the nutritional value of edible insects is highly variable. However, insects have significant protein content which varies from 20 to 76% of dry matter, depending on the type and development stage of the insect [19]. Therefore, edible insects may be a good source of bioactive peptides. There are many studies of the nutritional value of edible insects [20,21,22,23,24,25], but there are no data explaining the effect of the thermal process (e.g., boiling, baking) on the antioxidant and anti-inflammatory properties of edible insects. Moreover, most studies are based on whole insects caught in the wild. For these reasons, in this study three species of insects (*Tenebrio molitor*, *Schistocerca gregaria*, *Gryllodes sigillatus*) were selected, which are well known and whose breeding system is well-elaborated in Europe. *Tenebrio molitor* and *Gryllodes sigillatus* have also been reported to have the greatest potential to be used as food and feed in the EU [26].

The aim of this study was to determine the influence of heat treatment of edible insects (raw, boiled, baked) on antioxidant and anti-inflammatory activity of hydrolysates and peptide fractions obtained under simulated digestion and absorption gastrointestinal conditions. The antioxidant activity was expressed as free radical-scavenging activity, ion chelating activity, and reducing power, whereas the anti-inflammatory activity was determined as lipoxygenase and cyclooxygenase-2 inhibitory activity.

## 2. Materials and Methods

### 2.1. Samples

Three species of insects were investigated: mealworms *Tenebrio molitor* (Linnaeus, Coleoptera: Tenebrionidae) (larvae), locusts *Schistocerca gregaria* (Forskal, Orthoptera: Acrididae) (adult), and crickets *Gryllodes sigillatus* (Fabricius, Orthoptera: Gryllidae) (adult). Specimens of insects were obtained from a commercial supplier (Fabryka Owadów, Warsaw, Poland).

Wheat brans were used as a feed for mealworms (values for 100 g of product: energy: 775 kJ/185 kcal, fat 5.1 g, carbohydrates 19.5 g, of which sugars 2.2 g, fibre 42.4 g, protein 16.0 g. Crickets were fed with a compound feed (values provided by the breeder: protein 21%, fat 4%, fibre 6.5%). Wheat brans and grass were used as a feed for locusts.

### 2.2. Chemicals

TNBS (picrylsulfonic acid), linoleic acid, ferrozine (3-(2-pyridyl)-5,6-bis-(4-phenyl-sulfonic acid)-1,2,4-triazine), ABTS (2,2′-azino-bis(3-ethylbenzothiazoline-6-sulfonic acid)), DPPH (2,2-diphenyl-1-picrylhydrazyl), lipoxygenase (LOX) from Glycine max (cat no. L7395, >50,000 U/mg), α-amylase from hog pancreas (cat no. 10080, 50 U/mg), pepsin from porcine gastric mucosa (cat no. 10080, 250 U/mg), pancreatin from porcine pancreas (no cat. P1750), bile extract, and the molecular marker were purchased from Sigma-Aldrich (SigmaMarker, Sigma-Aldrich, St. Louis, MI, USA, cat no. S8445)). The COX Activity Assay Kit was purchased from Cayman Chemical (Cayman Chemical Company, Ann Arbor, MI, USA) (cat no. 701050). All of the other chemicals used were of analytical grade.

### 2.3. Methods of Edible Insect Preparation

All individuals of these species were fasted for approximately 48 h to clear their gastrointestinal tract of any residual food. Each species of insects tested was treated with heat. One of them was added to boiling water and boiled for 10 min at 100 °C. The others were baked in a heated oven at 150 °C for 10 min. For each species tested and type of thermal treatment (boiling, baking, and raw), approximately 0.5 kg of material was frozen, lyophilised (FreeZone 2.5, Labconco, Kansas City, KS, USA), and kept at −18 °C for further analysis.

### 2.4. The Method of Obtaining Protein Preparation

Proteins were isolated from the studied species of insects according to the method described by Girón-Calle, Alaiz, and Vioque [27] with slight modification [12] regarding the pH of solution. Briefly, raw insects were ground and mixed with 0.2% NaOH in a ratio of 1:10 (*w/v*). Protein extraction was carried out under stirring for 1 h at room temperature. After that, the samples were centrifuged at 4 °C for 20 min at 8000× *g*. pH of the obtained supernatants was adjusted to the isoelectric point of insect proteins with 0.1 M HCl. Precipitated proteins were centrifuged at 4 °C for 20 min at 8000× *g* and washed with distilled water. Protein preparations obtained from the insects were lyophilised and kept at −18 °C to further analysis.

### 2.5. SDS-Polyacrylamide Gel Electrophoresis Protein Profile

SDS-PAGE analysis of proteins from the insects was performed on 5% concentrated and 12% separated polyacrylamide gel. A Mini-Protean BioRad (BioRad, Hercules, CA, USA) electrophoresis system with 20 mA was used. The samples were mixed with buffer samples (BioRad, 1:1, *v/v*) and were heated at 100 °C for 5 min prior to the electrophoresis run. The gels were stained with Coomassie Blue R-250 using the molecular marker in the range of 6.5–200.0 kDa (SigmaMarker, Sigma-Aldrich) as a standard.

### 2.6. Enzymatic Hydrolysis and Absorption Process

In vitro digestion of lyophilisates from whole insects and insect protein was carried out according to the method described by Jakubczyk et al. [28] with slight modification. Briefly, samples were suspended (4%, *w/v*) in a stimulated saliva solution. Enzymatic hydrolysis was carried out at 37 °C in darkness with use of gastrointestinal enzymes (α-amylase, pepsin, pancreatin, and bile extract solution). The reaction was stopped by heating at 100 °C for 5 min. The hydrolysates were clarified by centrifugation at 8000× *g* for 10 min (MPW, 350R, Warsaw, Poland).

The hydrolysates were dialyzed with a membrane tube (molecular weight cut-off 3.5 kDa, Serva, Heidelberg, Germany). The absorption process was carried out without light for 1 h at 37 °C. After this stage, the samples were lyophilised and stored at −18 °C until further use.

### 2.7. Determination of the Peptide Concentration

The concentration of soluble peptides was determined with the trinitrobenzenesulphonic acid (TNBS) method using l-leucine as a standard [29].

### 2.8. Free Radical Scavenging Assay

#### 2.8.1. DPPH Radical Scavenging Activity Assay

DPPH radical (DPPH^•^) scavenging activity of the peptide fractions was analysed according to Brand-Williams, Cuvelier, and Berset [30] with slight modification [12] regarding quantities of antioxidant solution. A 0.04 mL volume of the sample was mixed with 0.96 mL of a 6 µM solution of DPPH^•^ in 75% methanol. The absorbance was measured after 3 min of the reaction at 515 nm. 75% methanol was used as a blank. The scavenging effect was calculated according to Equation (1):

(1) $$Scavenging\;activity\;\left(\%\right)\;=\;\left[1\;-\;\frac{Asample}{Acontrol}\right]\;\times \;100$$

where Asample is the absorbance of the DPPH^•^ solution with the sample; and Acontrol is the absorbance of the DPPH^•^ solution without the sample.

The results were calculated as the IC_50_ value (inhibitory concentration). The IC_50_ was determined by assesment of the free radical scavenging activity of several dilutions of each sample and interpolating the peptide concentration at which the inhibition percentage reached 50%. The IC_50_ value was calculated from the graph plotting scavenging activity for the four different contents of peptides.

#### 2.8.2. ABTS Radical Scavenging Activity Assay

ABTS radical cation (ABTS^•+^) scavenging activity was determined according to Re et al. [31] with slight modification [12] regarding the quantities of the antioxidant solution. The radical solution was prepared with ABTS and potassium persulfate, diluted in water at a final concentration of 2.45 mM, and left in the dark for 16 h to allow for radical development. The solution was diluted to reach the absorbance measures around 0.7 at 734 nm. 0.96 mL of the ABTS^•+^ solution was mixed with 0.04 mL of each sample. The absorbance was measured after 3 min of the reaction at 734 nm. Deionized water was used as a blank. The scavenging effect was calculated according to Equation (2):

(2) $$Scavenging\;activity\;\left(\%\right)\;=\;\left[1\;-\;\frac{Asample}{Acontrol}\right]\;\times \;100$$

where Asample is the absorbance of the ABTS^•+^ solution with the sample; and Acontrol is the absorbance of the ABTS^•+^ solution without the sample.

The results were calculated as the IC_50_ value (inhibitory concentration). The IC_50_ was determined by assessment of the free radical scavenging activity of several dilutions of each sample and interpolating the peptide concentration at which the inhibition percentage reached 50%. The IC_50_ value was calculated from the graph plotting scavenging activity for the four different contents of peptides.

### 2.9. Determination of Fe^2+^ Chelating Activity

The Fe^2+^ chelation assay was carried out according to the procedure described by Decker and Welch [32] with slight modification [12]. Briefly, 1 mL of sample was mixed with 0.02 mL of a 2 mM FeCl_2_ solution and 0.04 mL of 5 mM ferrozine. The mixture was shaken vigorously. The absorbance was measured at 562 nm after 10 min of incubation at room temperature. The chelating activity was calculated according to Equation (3):

(3) $$Chelation\;activity\;\left(\%\right)\;=\;\left[1\;-\;\frac{Asample}{Acontrol}\right]\;\times \;100$$

where Asample is the absorbance of the solution with the sample; and Acontrol is the absorbance of the solution without the sample.

The results were calculated as the IC_50_ value (inhibitory concentration). The IC_50_ was determined by assessment of the ion chelating activity of several dilutions of each sample and interpolating the peptide concentration at which the inhibition percentage reached 50%. The IC_50_ value was calculated from the graph plotting chelating activity for the four different content of peptides.

### 2.10. Ferric-Reducing Power

Reducing power was determined according to Hu, Song, and Gu [33] with some modifications [12] regarding the quantities of the reagents. A 0.96 mL volume of 0.2 M phosphate buffer (pH 6.6) and 1 mL of 1% potassium ferricyanide (*w/v*) were mixed with 1 mL of the sample. The mixture was incubated at 50 °C for 20 min. After that, 1 mL of 10% TCA (*w/w*), 1 mL of distilled water, and 0.2 mL of 0.1% ferric chloride (*w/v*) were added. The absorbance was read after a 10 min reaction at 700 nm.

### 2.11. Anti-Inflammatory Activity

#### 2.11.1. Lipoxygenase Inhibitory Activity Assay

Lipoxygenase (LOX) inhibitory activity was determined according to the procedure described by Axelroad, Cheesborough, and Laakso [34]. Lipoxygenase activity was determined spectrophotometrically by measuring the increase in absorbance at 234 nm over a 3 min period, at 25 °C. The reaction mixture contained 1 mL of 1/15 M phosphate buffer, pH 7.0, 10 µL of a lipoxygenase solution (167 U/mL), and 50 µL of an inhibitor solution. After pre-incubation of the mixture for 3 min, the reaction was initiated by adding 40 µL of 2.5 mmol/L linoleic acid. The corresponding control contained the same concentration of the enzyme without an inhibitor. The %inhibition was calculated according to Equation (4):

(4) $$\%Inhibition\;=\;\left[\frac{Activity\;of\;LOX\;-\;Activity\;of\;LOX\;with\;sample}{Activity\;of\;LOX}\right]\;\times \;100$$

The results were calculated as the IC_50_ value (Inhibitory Concentration). The IC_50_ was determined by assessment of the LOX inhibition of several dilutions of each sample and interpolating the peptide concentration at which the inhibition percentage reached 50%. The IC_50_ value was calculated from the graph plotting inhibition for the four different content of peptides.

#### 2.11.2. Cyclooxygenase Inhibitory Activity Assay

To determine cyclooxygenase 2 (COX-2) inhibition, a Cayman Chemical COX Colorimetric Inhibitor Screening Assay Kit was used (Cayman Chemical, Ann Arbor, MI, USA). The assay measures the peroxidase component of COXs. The peroxidase activity is assayed colourimetrically by monitoring the appearance of oxidized *N,N,N′,N′*-tetramethyl-p-phenylenediamine (TMPD) at 590 nm. The positive control (100% of COX-2 activity) contained 150 µL of 0.1 M Tris–HCl buffer (pH 8.0), 10 µL of heme, and 10 µL of the enzyme. The positive control and inhibitory samples containing 150 µL of buffer, 10 µL of heme, 10 µL of enzyme, and 10 µL of the sample solution were incubated for 5 min at 25 °C and the reaction was initiated by adding 20 µL of an arachidonic acid (AA) solution and 20 µL of a colorimetric substrate solution (TMPD). The samples were incubated for 2 min at 25 °C and the absorbance was read at 590 nm. The %inhibition was calculated according to Equation (5):

(5) $$\%Inhibition\;=\;\left[\frac{Activity\;of\;COX\;-\;Activity\;of\;COX\;with\;sample}{Activity\;of\;COX}\right]\;\times \;100$$

The results were calculated as the IC_50_ value (Inhibitory Concentration). The IC_50_ was determined by assessment of the COX inhibition of several dilutions of each sample and interpolating the peptide concentration at which the inhibition percentage reached 50%. The IC_50_ value was calculated from the graph plotting inhibition for the four different content of peptides.

### 2.12. Statistical Analysis

All assays were performed in triplicate. All data are presented as means ± standard deviation. Statistical analyses were carried out using the Statistica (version 10.0, StatSoft, Krakow, Poland). Tukey’s test was used to compare the groups. Differences between the mean values were found statistically significant at a *p* value less than 0.05.

## 3. Results and Discussion

### 3.1. Electrophoresis Profile

**Figure 1:**
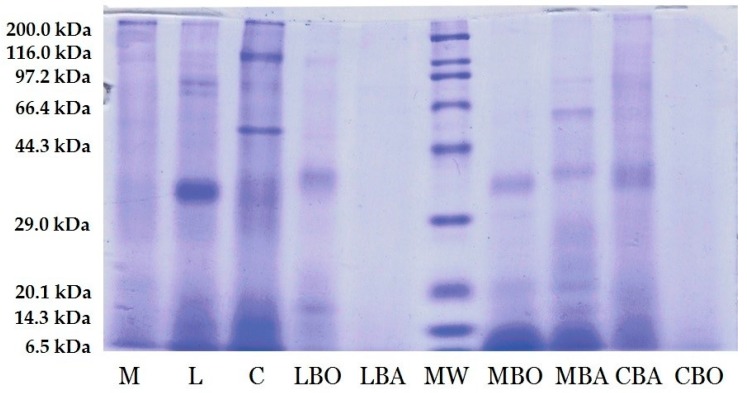
Electrophoretic (sodium dodecyl sulfate polyacrylamide gel electrophoresis) pattern of insect protein. Lane: M = raw mealworm *T. molitor*, L = raw locust *S. gregaria*, C = raw cricket *G. sigillatus*, LBO = boiled locust *S. gregaria*, LBA = baked locust *S. gregaria*, MBO = boiled mealworm *T. molitor*, MBA = baked mealworm *T. molitor*, CBA = baked cricket *G. sigillatus*, CBO = boiled cricket *G. sigillatus*, MW = molecular mass standard

The protein profile of the heat-treated insects was monitored by sodium dodecyl sulfate polyacrylamide gel electrophoresis (SDS-PAGE) (BioRad, Hercules, CA, USA). The heat treatment of insects resulted in disappearance of bands with high molecular mass and appearance of bands with lower mass. The raw, boiled, and baked insect proteins (Figure 1) exhibited bands in a range of 6.5–97.2 kDa. It should be noted that the SDS-PAGE profile in the raw crickets showed 116.0 kDa bands. The major bands were identified in a range between 29.0 and 44.3 kDa and at 66.4 kDa.

### 3.2. Peptide Concentration

**Figure 2:**
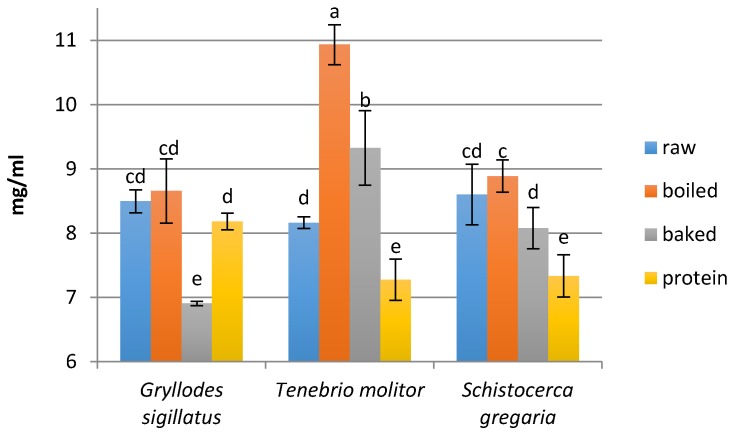
Peptide concentration after the hydrolysis process (mg/mL). Means ± standard deviation (SD) in triplicate. Different letters indicate significant difference (*p* < 0.05).

**Figure 3:**
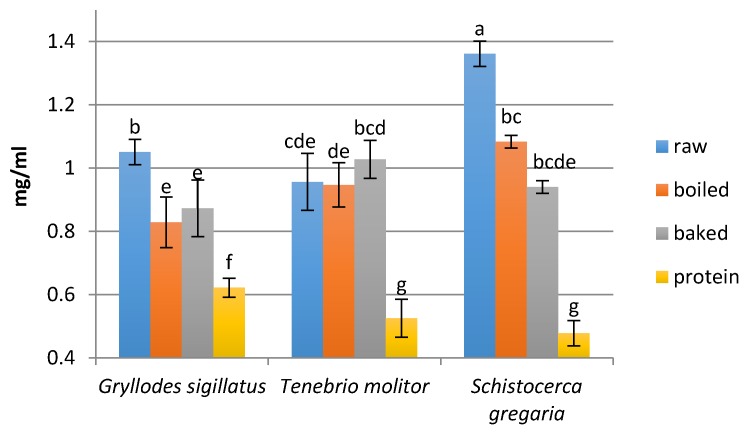
Peptide concentration after the in vitro absorption process (mg/mL). Means ± SD in triplicate. Different letters indicate significant difference (*p* < 0.05).

The edible insects were digested under simulated gastrointestinal conditions and a simulated absorption process was carried out next. In all samples, the concentration of peptides was decreased after the absorption process (Figure 2 and Figure 3), as the hydrolysates were dialyzed with a membrane tube (molecular weight cut-off 3.5 kDa). The highest concentration of peptides after digestion was noted for the boiled mealworm *Tenebrio molitor* (10.93 mg/mL), whereas the highest concentration of peptides after the absorption process was found in the fraction obtained from the raw locust *Schistocerca gregaria* hydrolysate (1.36 mg/mL). Generally, the highest amount of peptides was found in hydrolysates obtained by digestion of boiled insects and the lowest peptide concentration was noted for protein hydrolysates (except the hydrolysate from the baked cricket *Gryllodus sigillatus*). In the case of peptide fractions obtained after the in vitro absorption process of hydrolysates, the lowest amounts of peptides also was found in the samples obtained from the protein preparations, while the samples obtained from raw insects exhibited the highest values (except the baked *T. molitor*). Generally, the heat treatment caused a slight reduction in the content of peptides.

It should be noted that the content of peptides after the digestion of the studied edible insects was higher than the peptide content in similar insect hydrolysates: *Gryllodes sigillatus* 8.5 mg/mL and another cricket *Amphiacusta annulipes* 1.68 mg/mL, *Tenebrio molitor* 8.16 mg/mL and other larvae of the beetle *Zophobas morio* 1.88 mg/mL, *Schistocerca gregaria* 8.6 mg/mL and the other locust *Locusta migratoria* 5.88 mg/mL [6]. The concentration of peptides after the in vitro digestion of the edible insects was also higher than the peptide content after legume protein hydrolysis. Hydrolysate of fermented pea seeds was shown to have a 3.01 mg/mL peptide concentration [28].

### 3.3. Free Radical Scavenging Assay

The highest antiradical activity against ABTS^•+^ was noted for the hydrolysate obtained from the raw *T. molitor* (IC_50_ value 5.3 µg/mL) and that against DPPH^•^ for the hydrolysates obtained from the baked *G. sigillatus* as well as the *S. gregaria* protein preparation (IC_50_ value 28.5 µg/mL) (Table 1). In turn, the peptide fraction obtained from the baked *G. sigillatus* was found to have the strongest ABTS^•+^ and DPPH^•^ scavenging capacity (IC_50_ value 10.07 µg/mL and 10.9 µg/mL, respectively) (Table 2). The results of the present study show that heat treatment of insects increased the antiradical activity of hydrolysates and peptide fractions and particularly baking was characterized by better results. That is because during heat treatment, the sequence of insect peptide was changed [35]. According to the obtained results, it should be concluded that heat treatment process had effect on the release of peptides with antioxidant properties. The results presented by Karaś et al. [12] also suggest that the heat treatment has a positive impact on the antioxidant activity of chickpea seeds. Moreover, these results correspond well with those obtained for beef meat [36]. The DPPH^•^ scavenging activity of the extracted peptides from cooked meat was much higher than the scavenging activity of peptides from raw meat—even 20%. In turn, Serpen et al. [37] reported that it is possible that mild heat treatments could increase the antioxidant activity of some proteins from chicken, pork, and beef due to changes in their quaternary and tertiary structures. Furthermore, most frequently, samples after the absorption process were characterized by higher antiradical activity than hydrolysates. The lowest antiradical activity against ABTS^•+^ was obtained in the boiled *T. molitor* hydrolysate (IC_50_ value 28.9 µg/mL) and the activity against DPPH^•^ was the lowest in the raw *T. molitor* hydrolysate (IC_50_ value 109.4 µg/mL).

Higher antiradical activity against ABTS^•+^ was found in hydrolysate obtained after the digestion process of *Zophobas morio* (IC_50_ value 4.6 µg/mL) and the activity against DPPH^•^ was higher in hydrolysate obtained after digestion of the *Amphiacusta annulipes* (IC_50_ value 19.1 µg/mL) [5], whereas, the IC_50_ value for the antiradical activity against DPPH^•^ for the silkworm protein hydrolysate was estimated at 57.91 µg/mL [38]. The results by Bräunlich et al. [39] showed that the IC_50_ value of antiradical activity against DPPH^•^ of a 50% EtOH crude extract of aronia berries (*Aronia melanocarpa*) was estimated at 25 µg/mL. This it was a similar to the best results of the hydrolysates analysed in these studies (baked *G. sigillatus* and *S. gregaria* protein preparation hydrolysates—IC_50_ value 28.5 µg/mL). These results correspond well with those obtained for a water extract of fresh rose hips (*Rosa canina*) (IC_50_ value 32.7 µg/mL), which is known for its strong antioxidant properties [40]. Moreover, the investigated hydrolysates and the peptide fractions showed higher DPPH^•^ scavenging capacity than peptides obtained from loach hydrolysate. You et al. [35] reported the IC_50_ value as 1.65 mg/mL for peptides from loach hydrolysate after the heat treatment (20 min at 100 °C) when the concentration of loach peptides was 10 mg/mL.

The antioxidant activity of a peptide fraction (<3.5 kDa) obtained by in vitro gastrointestinal digestion of chickpea seed proteins were lower than in the peptide fractions obtained from the studied edible insects. The highest antiradical activity against ABTS^•+^ was observed for the peptide fraction obtained from heat-treated chickpea hydrolysate, and the IC_50_ value was 44.2 µg/mL, which is a substantially higher value than the lowest antiradical activity among the peptide fractions obtained from the studied species of insects (protein preparation from *T. molitor*—IC_50_ value 24.31 µg/mL). In the presented data, the IC_50_ value for the antiradical activity against DPPH^•^ was noted to be 55.48 µg/mL [12].

### 3.4. Ion Chelating Activity

The highest ability to chelate Fe^2+^ ions was shown by the *G. sigillatus* protein preparation hydrolysate among all the hydrolysates analysed (IC_50_ value 16.18 µg/mL) (Table 1) and the peptide fraction obtained from the boiled *S. gregaria* among the peptide fractions <3.5 kDa (IC_50_ value 2.57 µg/mL) (Table 2). Generally, the heat treatment of the insects increased the ability of the hydrolysates and peptide fractions to chelate Fe^2+^. The use of thermal processing significantly influences the release of biologically-active peptides during in vitro gasgastrointestinal digestion of proteins [12]. High chelating activity of insect peptides might be related to the specific peptide structure and amino acid side chain groups which played an important role not only in terminating the free radical chain reactions but also in chelating transitionmetal ions [41]. Furthermore, the highest ability to chelate Fe^2+^ was found most frequently in the baked insects. These results correspond well with those obtained by Karaś et al. [12]. They reported an IC_50_ value of 81.6 µg/mL for a peptide fraction <3.5 kDa from raw chickpea and 78.86 µg/mL for a peptide fraction from heat-treated chickpea. These IC_50_ values are higher than our results, but the heat treatment of chickpea also increased the ability of peptide fractions to chelate Fe^2+^ ions. In turn, different result was reported by Wu et al. [38] for silkworm protein hydrolysate with IC_50_ value determined to be 2.03 mg/mL.

### 3.5. Ferric-Reducing Power

A higher value of reducing power was noted for the hydrolysates than the peptide fractions. The highest value was found in the raw *G. sigillatus* hydrolysate (0.771), while the locust *S. gregaria* protein preparation hydrolysate showed the lowest value (0.364) (Table 1). The absorbance for the peptide fraction obtained from the boiled *G. sigillatus* was 0.298, but high values were also noted for the peptide fractions from the baked *G. sigillatus* and the baked *T. molitor* (0.286 and 0.280, respectively) (Table 2). Generally, the lowest results were noted for the protein preparations among the hydrolysates and for samples after the absorption process. Furthermore, it seems that heat treatment of insects contributes mainly to increased reducing power among peptide fractions.

The presented data correspond well with other results obtained for edible insects where the highest value of reducing power also was noted for cricket hydrolysate (*Amphiacusta annulipes*) 0.652. Similar results (0.522) were noted for hydrolysate from beetle larvae (*Zophobas morio*) as for the *T. molitor* hydrolysate (0.477). In contrast, the analysed locust species (*Schistocerca gregaria*) was characterized by higher reducing power than the other locust (*Locusta migratoria*)—0.533 and 0.210, respectively [6]. These results correspond well with those obtained by You et al. [35]. They reported the absorbance of 0.876, 0.511, and 0.487 for loach peptide after 15 min at 121 °C, 20 min at 100 °C, and 30 min at 63 °C thermal processing, respectively. Moreover, egg white protein powder hydrolysates were characterized by similar values (0.6) [42]. In turn, the reducing power for grass carp alcalase hydrolysate was only 0.19 [43]. Additionally, high reducing power (0.83) was detected for a silkworm protein hydrolysate [38]. Moreover, the investigated hydrolysates showed higher reducing power than medicinal plants, leafy vegetables or legumes such as *Moringa oleifera*, *Amaranthus viridis*, *Tamarindus indica*, *Spinacia oleracea*, and *Trigonella foenum-graecum* [16,44,45]. In turn, different results were reported by Carrasco-Castilla et al. [16] for peptide fractions >1 kDa from bean protein hydrolysates with absorbance determined to be 0.118. Similarly, Li et al. [46] also reported absorbance in a range between 0.12 and 0.3 for peptide fractions from chickpea protein hydrolysates. Additionally, the absorbance obtained for peptide fractions (1–3 kDa) from oyster meat was 0.4 [47].

### 3.6. Lipoxygenase and Cyclooxygenase-2 Inhibitory Activity

The conducted research proved that the activity of lipoxygenase (LOX) and cyclooxygenase-2 (COX-2) was effectively inhibited by the edible insect hydrolysates and peptide fractions (Table 1, Table 2). The *S. gregaria* protein hydrolysate was the most effective inhibitor of LOX (IC_50_ value 0.65 mg/ml), while the raw *S. gregaria* hydrolysate inhibited COX-2 most efficiently (IC_50_ value 10.91 µg/mL). The peptide fraction from the *S. gregaria* protein hydrolysate showed the best LOX and COX-2 inhibitory activity among the peptide fractions (IC_50_ value 3.13 µg/mL and 5.05 µg/mL, respectively). Generally, the best inhibitory activity of the LOX and COX (as anti-inflammatory activity) was determined for the peptide fractions and hydrolysates obtained after digestion of the insect protein preparations. Better inhibitory activity was noted for the peptide fractions than the hydrolysates.

In our research, edible insects showed high LOX and COX-2 inhibitory potential. Lipoxygenase inhibition by anthocyanins from purple basil leaves was found to have an IC_50_ value of 41.0 mg/g [46]. Moreover, similar results to our peptide fractions were noted for aronia extracts after fractionation (*Aronia melanocarpa*): the IC_50_ value was in the range between 30.3 and 91.0 µg/mL [39]. Furthermore, in the COX-2 inhibitory assay, the value for the peptide fraction from the *S. gregaria* protein preparation (IC_50_ value 5.05 µg/mL) is similar to that presented by Szymanowska et al. [48] for purple basil leaves (IC_50_ value 5.0 µg/mL). Additionally, the IC_50_ value obtained for rose hip (*Rosa canina*) was in the range between 19.0 and 62.0 µg/mL, which is similar to our result obtained for the selected hydrolysates and peptide fractions, for example, the protein fractions obtained from the boiled *G. sigillatus* hydrolysate and boiled *S. gregaria* hydrolysate (IC_50_ value 18.71 µg/mL and 20.92 µg/mL, respectively), or boiled *S. gregaria* hydrolysate (IC_50_ value 61.6 µg/mL) [49].

## 4. Conclusions

It can be concluded that edible insects are a rich source of bioactive peptides with antioxidant and anti-inflammatory activities. Our results show that, after the digestion and absorption process, edible insects have high antiradical activity and an ability to chelate iron ions and can inhibit lipoxygenase and cyclooxygenase-2 activity. Moreover, the heat treatment process positively affects the antioxidant properties of peptides. These results indicates that the technology process involving heat treatment has a significant effect on the accessibility of proteins for enzymatic digestion, leading to the increase in antioxidant peptide content. It should be noted that consumption of edible insects and foods enriched with insect proteins or fats may be potentially beneficial to the human body. Entomophagy may be very well proved to be a good idea in preventing civilization diseases, as edible insects may potentially prevent diseases associated with oxidative stress.

## Figures and Tables

**Table 1:** The antioxidant and anti-inflammatory activities of hydrolysates.

Studied Species	Type of Heat Treatment	2,2′-Azino-bis(3-ethylbenzothiazoline-6-sulfonic Acid) Radical Cation (ABTS^•+^) Scavenge (IC_50_ µg/mL)	2,2-Diphenyl-1-picrylhydrazyl Radical (DPPH^•^) Scavenge (IC_50_ µg/mL)	Fe^2+^ Chelating Activity (IC_50_ µg/mL)	Reducing Power (Abs_700_)	Lipoxygenase (LOX) Inhibitory Activity (IC_50_ mg/mL)	Cyclooxygenase (COX) Inhibitory Activity (IC_50_ µg/mL)
*Gryllodes sigillatus*	raw	21.9 ± 1.13 ^d^	29.45 ± 1.63 ^h^	85.64 ± 1.02 ^a^	0.771 ± 0.02 ^a^	3.14 ± 0.06 ^a^	69.91 ± 0.4 ^h^
	boiled	21.8 ± 0.71 ^d^	40.3 ± 0.57 ^g^	53.97 ± 0.88 ^f^	0.611 ± 0.02 ^b^	2.04 ± 0.05 ^c^	146.8 ± 1.6 ^c^
	baked	17.5 ± 0.57 ^f^	28.5 ± 0.57 ^i^	38.6 ± 0.6 ^j^	0.588 ± 0.02 ^bc^	2.24 ± 0.02 ^bc^	38.51 ± 0.84 ^k^
	protein	21.65 ± 0.47 ^de^	29.5 ± 1.6 ^h^	16.18 ± 0.1 ^k^	0.551 ± 0.01 ^bcd^	0.79 ± 0.02 ^f^	208.24 ± 1.6 ^a^
*Tenebrio molitor*	raw	5.3 ± 0.42 ^j^	109.4 ± 1.13 ^a^	67.52 ± 0.8 ^c^	0.477 ± 0.04 ^defg^	1.57 ± 0.04 ^d^	58.09 ± 1.24 ^j^
	boiled	28.9 ± 1.41 ^a^	97.45 ± 1.62 ^c^	69.71 ± 0.69 ^b^	0.566 ± 0.04 ^bcde^	1.59 ± 0.04 ^d^	108.51 ± 2.22 ^d^
	baked	24.5 ± 1.27 ^c^	85.85 ± 2.33 ^e^	52.14 ± 0.74 ^g^	0.504 ± 0.03 ^def^	2.29 ± 0.06 ^b^	82.59 ± 2.11 ^g^
	protein	21.4 ± 0.63 ^e^	40.3 ± 0.6 ^g^	60.78 ± 0.97 ^d^	0.373 ± 0.06 ^h^	0.89 ± 0.02 ^f^	92.06 ± 1.2 ^f^
*Schistocerca gregaria*	raw	27.5 ± 0.99 ^b^	79.5 ± 1.27 ^f^	58.21 ± 1.02 ^e^	0.533 ± 0.02 ^bcde^	1.3 ± 0.02 ^e^	10.91 ± 0.4 ^l^
	boiled	8.4 ± 0.85 ^h^	104.5 ± 3.39 ^b^	60.31 ± 0.87 ^d^	0.403 ± 0.02 ^gh^	1.28 ± 0.02 ^e^	61.6 ± 2.16 ^i^
	baked	6.9 ± 1.13 ^i^	91.1 ± 1.98 ^cd^	51.19 ± 0.65 ^h^	0.452 ± 0.02 ^efgh^	0.83 ± 0.02 ^f^	198.51 ± 3.16 ^b^
	protein	16.6 ± 0.86 ^g^	28.5 ± 0.6 ^i^	43.13 ± 0.99 ^i^	0.364 ± 0.02 ^fgh^	0.65 ± 0.02 ^f^	98.13 ± 2.14 ^e^

Means ± standard deviation (SD) in triplicate. Different letters in the same column indicate significant difference (*p* < 0.05).

**Table 2:** The antioxidant and anti-inflammatory activities of samples after the in vitro absorption process.

Studied Species	Type of Heat Treatment	ABTS^•+^ Scavenge (IC_50_ µg/mL)	DPPH^•^ Scavenge (IC_50_ µg/mL)	Fe^2+^ Chelating Activity (IC_50_ µg/mL)	Reducing Power (Abs_700_)	LOX Inhibitory Activity (IC_50_ µg/mL)	COX Inhibitory Activity (IC_50_ µg/mL)
*Gryllodes sigillatus*	raw	15.2 ± 1.83 ^g^	12.35 ± 0.07 ^j^	54.78 ± 1.12 ^c^	0.255 ± 0.07 ^bc^	141.81 ± 2.43 ^b^	16.34 ± 0.21 ^e^
	boiled	12.6 ± 3.04 ^i^	11.85 ± 0.21 ^k^	27.23 ± 0.88 ^d^	0.298 ± 0.04 ^a^	187.44 ± 2.11 ^a^	18.71 ± 0.2 ^d^
	baked	10.7 ± 0.71 ^k^	10.9 ± 0.14 ^l^	17.8 ± 0.65 ^f^	0.286 ± 0.09 ^a^	103.15 ± 1.65 ^d^	12.64 ± 0.45 ^f^
	protein	15.24 ± 1.7 ^g^	17.97 ± 0.05 ^i^	63.42 ± 2.01 ^a^	0.228 ± 0.03 ^cde^	6.95 ± 0.1 ^j^	9.89 ± 0.12 ^h^
*Tenebrio molitor*	raw	18.1 ± 1.13 ^e^	59.5 ± 1.98 ^e^	19.5 ± 0.89 ^e^	0.238 ± 0.01 ^cd^	94.68 ± 0.42 ^e^	9.88 ± 0.32 ^h^
	boiled	19.7 ± 1.55 ^d^	18.4 ± 1.83 ^h^	11.8 ± 0.54 ^g^	0.202 ± 0.02 ^ef^	62.17 ± 0.54 ^g^	11.33 ± 0.45 ^g^
	baked	13.1 ± 1.41 ^h^	25.1 ± 1.55 ^g^	9.93 ± 0.1 ^h^	0.280 ± 0.02 ^ab^	38.4 ± 0.2 ^i^	11.46 ± 0.3 ^g^
	protein	24.31 ± 1.88 ^a^	85.55 ± 1.83 ^b^	12.32 ± 0.21 ^g^	0.203 ± 0.01 ^ef^	3.82 ± 0.12 ^k^	9.53 ± 0.15 ^i^
*Schistocerca gregaria*	raw	21.5 ± 1.4 ^b^	81.4 ± 1.4 ^d^	57.97 ± 0.64 ^b^	0.204 ± 0.02 ^ef^	133.4 ± 0.96 ^c^	26.37 ± 0.45 ^a^
	boiled	20.2 ± 1.6 ^c^	81.8 ± 1.7 ^c^	2.57 ± 0.05 ^i^	0.197 ± 0.07 ^f^	80.62 ± 1.67 ^f^	20.92 ± 0.2 ^c^
	baked	17.8 ± 1.1 ^f^	29.1 ± 1.4 ^f^	11.93 ± 0.11 ^g^	0.211 ± 0.04 ^def^	49.57 ± 1.12 ^h^	23.82 ± 0.45 ^b^
	protein	12.1 ± 0.01 ^j^	88.81 ± 3.71 ^a^	10.30 ± 0.2 ^h^	0.207 ± 0.01 ^ef^	3.13 ± 0.23 ^l^	5.05 ± 0.32 ^j^

Means ± SD in triplicate. Different letters in the same column indicate significant difference (*p* < 0.05).

## References

[1] Rumpold B.A., Schlüter O.K. (2013). Potential and challenges of insects as an innovative source for food and feed production. Innov. Food Sci. Emerg. Technol..

[2] Van der Spiegel M., Noordam M.Y., Van der Fels-Klerx H.J. (2013). Safety of novel protein sources (insects, microalgae, seaweed, duckweed, and rapeseed) and legislative aspects for their application in food and feed production. Compr. Rev. Food Sci. Food Saf..

[3] Van Huis A., Itterbeeck J.V., Klunder H., Mertens E., Halloran A., Muir G., Vantomme P. (2013). Edible Insects: Future Prospects for Food and Feed Security.

[4] Megido R.C., Gierts C., Blecker C., Brostaux Y., Haubruge É., Alabi T., Francis F. (2016). Consumer acceptance of insect-based alternative meat products in Western countries. Food Qual. Prefer..

[5] Verkerk M.C., Tramper J., Van Trijp J.C.M., Martens D.E. (2007). Insect cells for human food. Biotechnol. Adv..

[6] Zielińska E., Karaś M., Jakubczyk A. (2017). Antioxidant activity of predigested protein obtained from a range of farmed edible insects. Int. J. Food Sci. Technol..

[7] Gobbetti M., Stepaniak L., De Angelis M., Corsetti A., Di Cagno R. (2002). Latent bioactive peptides in milk proteins: Proteolytic activation and significance in dairy processing. Crit. Rev. Food Sci. Nutr..

[8] Saiga A., Tanabe S., Nishimura T. (2003). Antioxidant activity of peptides obtained from porcine myofibrillar proteins by protease treatment. J. Agric. Food Chem..

[9] Zhang M., Mu T.H., Wang Y.B., Sun M.J. (2012). Evaluation of free radical-scavenging activities of sweet potato protein and its hydrolysates as affected by single and combination of enzyme systems. Int. J. Food Sci. Technol..

[10] Karaś M., Jakubczyk A., Szymanowska U., Złotek U., Zielińska E. (2017). Digestion and bioavailability of bioactive phytochemicals. Int. J. Food Sci. Technol..

[11] Torres-Fuentes C., Alaiz M., Vioque J. (2011). Affinity purification and characterisation of chelating peptides from chickpea protein hydrolysates. Food Chem..

[12] Karaś M., Baraniak B., Rybczyńska K., Gmiński J., Gaweł-Bęben K., Jakubczyk A. (2015). The influence of heat treatment of chickpea seeds on antioxidant and fibroblast growth-stimulating activity of peptide fractions obtained from proteins digested under simulated gastrointestinal conditions. Int. J. Food Sci. Technol..

[13] Megías C., Pedroche J., Yust M.M., Girón-Calle J., Alaiz M., Millán F., Vioque J. (2007). Affinity purification of copper chelating peptides from chickpea protein hydrolysates. J. Agric. Food Chem..

[14] Stadtman E.R. (2006). Protein oxidation and aging. Free Radic. Res..

[15] Ali S.S., Kasoju N., Luthra A., Singh A., Sharanabasava H., Sahu A., Bora U. (2008). Indian medicinal herbs as sources of antioxidants. Food Res. Int..

[16] Carrasco-Castilla J., Hernández-Álvarez A.J., Jiménez-Martínez C., Jacinto-Hernández C., Alaiz M., Girón-Calle J., Vioque J., Dávila-Ortiz G. (2012). Antioxidant and metal chelating activities of *Phaseolus vulgaris* L. var. Jamapa protein isolates, phaseolin and lectin hydrolysates. Food Chem..

[17] Martel-Pelletier J., Lajeunesse D., Reboul P., Pellietier J.P. (2003). Therapeutic role of dual inhibitors of 5-LOX and COX, selective and non-selective non-steroidal anti-inflammatory drugs. Ann. Rheum. Dis..

[18] Shen F.Q., Wang Z.C., Wu S.Y., Ren S.Z., Man R., Wang B.Z., Zhu H.L. (2017). Synthesis of novel hybrids of pyrazole and coumarin as dual inhibitors of COX-2 and 5-LOX. Bioorg. Med. Chem. Lett..

[19] Zielińska E., Baraniak B., Karaś M., Rybczyńska K., Jakubczyk A. (2015). Selected species of edible insects as a source of nutrient composition. Food Res. Int..

[20] Finke M.D. (2012). Complete nutrient composition of commercially raised invertebrates used as food for insectivores. Zoo Biol..

[21] Ramos-Elorduy J. (2008). Energy supplied by edible insects from Mexico and their nutritional and ecological importance. Ecol. Food Nutr..

[22] Ramos-Elorduy J., Moreno J.M.P., Camacho V.H.M. (2012). Could grasshoppers be a nutritive meal?. Food Nutr. Sci..

[23] Rumpold B.A., Schlüter O.K. (2013). Nutritional composition and safety aspects of edible insects. Mol. Nutr. Food Res..

[24] Mlcek J., Rop O., Borkovcova M., Bednarova M. (2014). A comprehensive look at the possibilities of edible insects as food in Europe—A Review. Pol. J. Food Nutr. Sci..

[25] Adámková A., Adámek M., Mlček J., Borkovcová M., Bednářová M., Kouřimská L., Skácel J., Vítová E. (2017). Welfare of the mealworm (*Tenebrio molitor*) breeding with regard to nutrition value and food safety. Potr. Slovak J. Food Sci..

[26] EFSA Scientific Committee (EFSA) (2015). Risk profile related to production and consumption of insects as food and feed. EFSA J..

[27] Girón-Calle J., Alaiz M., Vioque J. (2010). Effect of chickpea protein hydrolysates on cell proliferation and in vitro bioavailability. Food Res. Int..

[28] Jakubczyk A., Karaś M., Baraniak B., Pietrzak M. (2013). The impact of fermentation and in vitro digestion on formation angiotensin converting enzyme (ACE) inhibitory peptides from pea proteins. Food Chem..

[29] Adler-Nissen J. (1979). Determination of the degree of hydrolysis of food protein hydrolysates with trinitrobenzenesulfonic acid. J. Agric. Food Chem..

[30] Brand-Williams W., Cuvelier M., Berset C. (1995). Use of a free radical method to evaluate antioxidant activity. LWT Food Sci. Technol..

[31] Re R., Pellegrini A., Proteggente A., Pannala M., Yang M., Rice-Evans C. (1999). Antioxidant activity applying an improved ABTS radical cation decolorization assay. Free Radic. Biol. Med..

[32] Decker E.A., Welch B. (1990). Role of ferritin as a lipid oxidation catalyst in muscle food. J. Agric. Food Chem..

[33] Hu L., Song R., Gu Z. (2012). An antioxidant peptide produced by autolysis reactions from wheat germ. Afr. J. Biotechnol..

[34] Axelroad B., Cheesborough T.M., Laakso S. (1981). Lipoxygenases in soybeans. Methods Enzymol..

[35] You L., Zheng L., Regenstein J.M., Zhao M., Liu D. (2012). Effect of thermal treatment on the characteristic properties of loach peptide. Int. J. Food Sci. Technol..

[36] Fu Y., Young J.F., Therkildsen M. (2017). Bioactive peptides in beef: Endogenous generation through postmortem aging. Meat Sci..

[37] Serpen A., Gökmen V., Fogliano V. (2012). Total antioxidant capacities of raw and cooked meats. Meat Sci..

[38] Wu Q.Y., Jia J.Q., Tan G.X., Xu J.L., Gui Z.Z. (2011). Physicochemical properties of silkworm larvae protein isolate and gastrointestinal hydrolysate bioactivities. Afr. J. Biotechnol..

[39] Bräunlich M., Slimestad R., Wangensteen H., Brede C., Malterud K.E., Barsett H. (2013). Extracts, anthocyanins and procyanidins from Aronia melanocarpa as radical scavengers and enzyme inhibitors. Nutrients.

[40] Nađpal J.D., Lesjak M.M., Šibul F.S., Anačkov G.T., Četojević-Simin D.D., Mimica-Dukić N.M., Beara I.N. (2016). Comparative study of biological activities and phytochemical composition of two rose hips and their preserves: Rosa canina L. and Rosa arvensis Huds. Food Chem..

[41] Zhu L.J., Chen J., Tang X.Y., Xiong Y.L. (2008). Reducing, radical scavenging, and chelation properties of in vitro digests of alcalase-treated zein hydrolysate. J. Agric. Food Chem..

[42] Lin S., Jin Y., Liu M., Yang Y., Zhang M., Guo Y., Jones G., Liu J., Yin Y. (2013). Research on the preparation of antioxidant peptides derived from egg white with assisting of high-intensity pulsed electric field. Food Chem..

[43] Li X., Luo Y., Shen H., You J. (2012). Antioxidant activities and functional properties of grass carp (*Ctenopharyngodon idellus*) protein hydrolysates. J. Sci. Food Agric..

[44] Raghavendra M., Reddy A.M., Yadav P.R., Raju A.S., Kumar L.S. (2013). Comparative studies on the in vitro antioxidant properties of methanolic leafy extracts from six edible leafy vegetables of India. Asian J. Pharm. Clin. Res..

[45] Mukherjee S., Pawar N., Kulkarni O., Nagarkar B., Thopte S., Bhujbal A., Pawar P. (2011). Evaluation of free-radical quenching properties of standard Ayurvedic formulation Vayasthapana Rasayana. BMC Complement. Altern. Med..

[46] Li Y., Jiang B., Zhang T., Mu W., Liu J. (2008). Antioxidant and free radical-scavenging activities of chickpea protein hydrolysate (CPH). Food Chem..

[47] Dong X.P., Zhu B.W., Zhao H.X., Zhou D.Y., Wu H.T., Yang J.F., Li D.M., Murata Y. (2010). Preparation and in vitro antioxidant activity of enzymatic hydrolysates from oyster (*Crassostrea talienwhannensis*) meat. Int. J. Food Sci. Technol..

[48] Szymanowska U., Złotek U., Karaś M., Baraniak B. (2015). Anti-inflammatory and antioxidative activity of anthocyanins from purple basil leaves induced by selected abiotic elicitors. Food Chem..

[49] Jäger A.K., Eldeen I.M.S., van Staden J. (2007). COX-1 and -2 Activity of Rose Hip. Phytother. Res..

